# OA28 A Joint Approach

**DOI:** 10.1093/rap/rkag113.028

**Published:** 2026-11-03

**Authors:** Sarah Bethel, Maria Hall, Lauren Huckerby, Emily Willis

**Affiliations:** Royal Manchester Children’s Hospital, Manchester, United Kingdom; Royal Manchester Children’s Hospital, Manchester, United Kingdom; Royal Manchester Children’s Hospital, Manchester, United Kingdom; Royal Manchester Children’s Hospital, Manchester, United Kingdom

## Abstract

**Introduction:**

We describe a case of an adolescent with polyarthritis and severe inflammatory bowel disease requiring ileostomy. Further investigations revealed a genetic diagnosis of RELA Associated Inflammatory Disease (RAID).

RAID is an autosomal dominant disorder caused by mutations in the RELA (p65) protein. RELA is involved in transcription of genes responsible for regulating cell apoptosis and inflammation. Whilst normally bound to inhibitory proteins (NFkB) within cell cytoplasm, stimuli such as TNF-alpha cause RELA to dissociate, binding to certain genes leading to their expression. In RAID, dysregulation in this pathway reduces cell survival, causing symptoms such as mucosal ulceration, arthritis and colitis.

**Case description:**

A 15 year old female was diagnosed 2 years ago with perianal Crohn’s disease after presenting with rectal bleeding and perianal ulceration. She received exclusive enteral nutrition induction and commenced Azathioprine and Infliximab. Due to non-response and high infliximab-antibody levels, alternate therapy with adalimumab was commenced. Azathioprine continued.

One year after Crohn’s diagnosis, she was referred to paediatric Rheumatology with joint pain. She had severe polyarthritis of knees, wrists, multiple MCP joints and right temporomandibular joint. Scaly plaques were noted to elbows and scalp, seen as psoriatic in nature, and a family history of psoriasis was noted. The patient reported recurrent oral ulcers, no history of genital ulcers. Bloods showed active inflammation; Erythrocyte sedimentation rate (ESR) 128, C-reactive protein (CRP) 84, HLA-B27 positive. Clinical diagnosis of polyarticular JIA associated with inflammatory bowel disease (IBD) was made.

A joint decision between rheumatology and gastroenterology was made to pulse with IV methylprednisolone followed by weaning oral prednisolone. Adalimumab was continued due to its efficacy in treating polyarthritis and IBD. The arthritis was responsive to high dose steroids but recurred once weaned.

Despite maintenance treatment with Azathioprine and weekly Adalimumab, the arthritis flared. Azathioprine was switched to methotrexate, and she received steroid joint injections. The patient developed anti-adalimumab antibodies.

6 months after commencing adalimumab, she attended gastroenterology clinic with worsening perianal symptoms. Examination found a large perianal fissure with open ulceration requiring hospital admission. Following surgical review and MDT discussion, she proceeded to have ileostomy formation.

MDT discussion took place between Gastroenterology, Rheumatology and Immunology given the severity of perianal disease, refractory to two anti-TNF medications. Differential diagnoses including chronic granulomatous disease, Behcet’s disease or mycobacterial infection were considered. Genetic R15 panel was sent and JAK-inhibitor Upadacitinib was commenced.

R15 genetic panel revealed a genetic diagnosis of RELA-associated inflammatory disease.

**Discussion:**

RAID is an interesting condition with only 7 cases identified in UK literature. While most autoinflammatory disease is driven by an increase in the NF-kB protein, this mutation results in reduced functional protein and impaired NF-kB activation, leading to TNF driven apoptosis in the mucosa and skin.

In RAID, the literature describes an overlap of symptoms with Bechet’s disease. In our case, predominant symptoms were arthritis, peri-anal and oral ulceration, with normal ophthalmological assessment. This patient had been managed with biologic therapy for almost a year prior to rheumatology assessment but despite this had significant active polyarthritis. As arthritis and IBD commonly overlap, this highlights the importance of early rheumatology review and effective multidisciplinary management with shared patient care. Managing this case required seamless communication with the gastroenterology team and additional MDT including specialist nurses, physiotherapy and occupational therapy.

As this patient is HLA-B27 positive with a family history of psoriasis, her initial presentation appeared to be supportive of classical IBD with an extra-intestinal feature of arthritis. However, it is important to note the atypical features at the time, with initial histology showing anal tissue with active inflammation and multiple necrotising epithelioid granulomas: TB was considered and ruled out at presentation.

As the patient journey evolved, her symptoms progressed and became refractory to standard treatment. This highlights the importance of prompt diagnostic re-evaluation and further investigation in patients who do not follow a typical trajectory. Similarly, her lead clinicians were unaware of the severity of her perianal symptoms emphasising the importance of a developmentally-appropriate approach in our adolescent consultations. This includes being mindful of privacy, lone consultation (at least for part of the review) and utilising developmentally appropriate tools e.g. HEADSSS (Home, Education, Activities, Drugs, Sexuality and relationships, Suicide and Mood, Safety) for adolescent history taking.

**Key learning points:**

• Re-evaluation of diagnosis should always be considered in refractory disease, or in patients who do not follow a typical trajectory.

• Consider genetic evaluation particularly in younger patients and those with difficult to control symptoms and multiple failed immunosuppressants.

• Implications for families: Consider genetic testing for families of RAID patients. One study noted similar symptoms in family members in patients with confirmed RAID.

• RAID is a monogenic mimic of Bechet’s disease with an overlap of symptoms. This has implications for treatment options e.g. colchicine and consideration should be given to undertake R15 genetic panel in all paediatric patients with suspected Bechet’s

• Importance of having an early rheumatology review in patients known to gastroenterology who have symptoms and signs of arthritis or autoinflammatory disease. Taking an MDT approach and joint care between specialities.

• Importance of developmentally appropriate care for adolescents, being mindful of privacy, lone consultation and appropriate tools for adolescent history taking.

